# Supplementary material for: The interactome of intact mitochondria by cross-linking mass spectrometry provides evidence for coexisting respiratory supercomplexes
Source: Mol Cell Proteomics. 2017 Dec 8;17(2):216–32. doi: 10.1074/mcp.RA117.000470 (PMC5795388; doi:10.1074/mcp.RA117.000470)
Supplement: Supplemental Data [file supp_RA117.000470_133922_0_supp_23978_fzffwf.zip › spectra_annotation/mito_spectra_annotation/254-1-12-1-18-1.pdf]

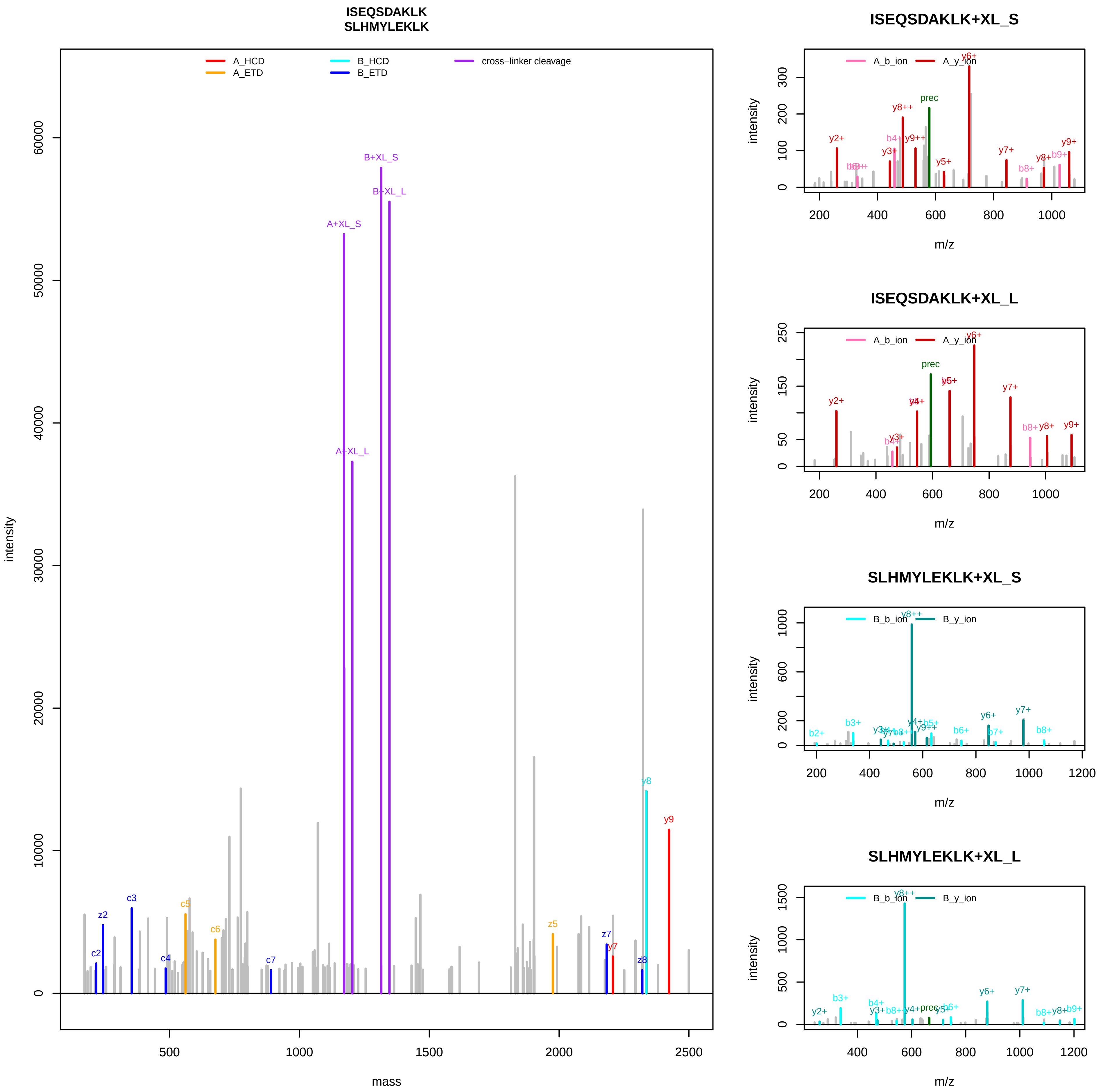
ISEQSDAKLK
SLHMYLEKLK
A_HCD
A_ETD
B_HCD
B_ETD
cross−linker cleavage
B+XL_S
B+XL_L
A+XL_S
A+XL_L
y8
y9
c2
z2
c3
c4
c5
c6
c7
z5
z7
y7
z8
intensity
mass
ISEQSDAKLK+XL_S
A_b_ion
A_y_ion
y6+
prec
y8++
y2+
y3+
y9++
y5+
y7+
y8+
b8+
b9+
y9+
b4+
m/z
ISEQSDAKLK+XL_L
A_b_ion
A_y_ion
y6+
prec
y5+
y2+
y4+
y7+
y3+
b8+
y8+
y9+
m/z
SLHMYLEKLK+XL_S
B_b_ion
B_y_ion
y8++
b2+
b3+
y3+
y4+
b5+
y9++
b6+
y6+
y7+
b7+
b8+
m/z
SLHMYLEKLK+XL_L
B_b_ion
B_y_ion
y8++
y2+
b3+
b4+
y3+
y4+
prec
y5+
b6+
y6+
y7+
b8+
y8+
b9+
m/z
